# The devil is in the detail - a multifactorial intervention to reduce blood pressure in co-existing diabetes and chronic kidney disease: a single blind, randomized controlled trial

**DOI:** 10.1186/1471-2296-11-3

**Published:** 2010-01-12

**Authors:** Allison F Williams, Elizabeth Manias, Rowan G Walker

**Affiliations:** 1 Melbourne School of Health Sciences, The University of Melbourne, Level 5, 234 Queensberry Street, Carlton, Australia 3053; 2 Department of Medicine, The University of Melbourne, Carlton, Australia 3053; 3 Nephrology Department, Royal Melbourne Hospital, Parkville, Australia

## Abstract

**Background:**

About 30-60% of individuals are non-adherent to their prescribed medications and this risk increases as the number of prescribed medications increases. This paper outlines the development of a consumer-centred *Me*dicine *S*elf-*M*anagement *I*ntervention (MESMI), designed to improve blood pressure control and medication adherence in consumers with diabetes and chronic kidney disease recruited from specialist outpatients' clinics.

**Methods:**

We developed a multifactorial intervention consisting of Self Blood Pressure Monitoring (SBPM), medication review, a twenty-minute interactive Digital Versatile Disc (DVD), and follow-up support telephone calls to help consumers improve their blood pressure control and take their medications as prescribed. The intervention is novel in that it has been developed from analysis of consumer and health professional views, and includes consumer video exemplars in the DVD. The primary outcome measure was a drop of 3-6 mmHg systolic blood pressure at three months after completion of the intervention. Secondary outcome measures included: assessment of medication adherence, medication self-efficacy and general wellbeing. Consumers' adherence to their prescribed medications was measured by manual pill count, self-report of medication adherence, and surrogate biochemical markers of disease control.

**Discussion:**

The management of complex health problems is an increasing component of health care practice, and requires interventions that improve patient outcomes. We describe the preparatory work and baseline data of a single blind, randomized controlled trial involving consumers requiring cross-specialty care with a follow-up period extending to 12 months post-baseline.

**Trial Registration:**

The trial was registered with the Australian and New Zealand Clinical Trials Register (ACTRN12607000044426).

## Background

### Comorbidity burden

The prevalence of chronic diseases continues to increase, necessitating sustained self-management of medications by consumers in the community and long term monitoring by health professionals. Diabetes and chronic kidney disease are two such rapidly escalating global health problems [1], and diabetes is now the most common cause of chronic kidney disease [2]. Irrespective of the cause of chronic kidney disease, the co-existence of diabetes and chronic kidney disease presents a significant health, social and economic burden and negatively influences health status beyond the sum of the effects of each disease: mortality is higher, quality of life is worse, and the burden on health care services is increased [3].

The complexity of the treatment for co-existing diabetes and chronic kidney disease involves comorbidity management, in particular hypertension, to improve disease control and health outcomes. Accordingly, the consumer is required to consult with numerous health professionals in primary and specialist settings who prescribe multiple medications for consumers to manage their health. Taking medications as prescribed is a principal requirement for well-managed chronic diseases. When multiple treatments are required for different health problems, medication-related concerns such as adherence, drug interactions and side effects, can be perceived as more troublesome to consumers than the treatments themselves [4,5].

Adherence can be defined as the extent to which consumers follow the instructions they are given for prescribed treatments [6]. Approximately 50% of consumers do not take their medications as prescribed. Interventions to assist medication adherence in the presence of co-existing chronic conditions have rarely been examined in past work and this research is of a high priority [7,8]. The management of comorbidities is an increasing feature of health care practice.

While medical interventions are very important to manage and prevent further health problems in people with co-existing diabetes and chronic kidney disease, long-term health outcomes depend on the effectiveness of the choices that consumers make for themselves on a daily basis [9]. The quality of shared decision making between health professionals is critical to this process. Medication mismanagement and safety are a cause of great stress and cost to healthcare providers worldwide [10,11]: 3% of all hospital admissions relate to over-use, under-use and inappropriate use of medications [10]. Poorly controlled diabetes and chronic kidney disease lead to blood vessel injury resulting in heart failure, loss of vision, total kidney failure, and limb loss that disrupt the consumer's ability to engage in independent activities of daily living. This paper outlines the methodology of a clinical trial using the CONSORT Statement [12], which is designed to help consumers with diabetes and chronic kidney disease control their blood pressure and to take their medications as prescribed.

### Conceptual framework

The conceptual framework underpinning this project is the modified Health Belief Model (HBM) [13,14]. The model explains short- and long-term health behaviours by focusing on the attitudes and beliefs of individuals, and their perceived threat and net benefits of taking positive health-related action. The modified HBM is based on the understanding that people will take a recommended health action if they believe the action will avoid a negative health condition and they have the confidence or self-efficacy to undertake the action. The five concepts of the modified HBM include: perceived susceptibility to a particular health problem, the severity of the health problem, the benefits and barriers to taking positive health-related action, and self-efficacy [15]. These concepts account for people's readiness to act. The HBM model encompasses modifying or enabling psychosocial influences that affect a consumer's adherence to recommended health actions.

## Methods

### Overview of the study

This trial was designed to improve medication self-management in consumers with co-existing diabetes and chronic kidney disease by testing a multifactorial, consumer-centred intervention to improve systolic blood pressure control and medication adherence in a randomized controlled trial (RCT). Consumer-centred interactive interventions to aid decision-making are an effective way of helping consumers with diverse backgrounds to understand their chronic diseases and complex medication regimens [16].

### Study design

The study is a single-site, single-blind, longitudinal RCT. Participants have been allocated to one of two groups: the intervention group receives a multifactorial intervention designed to improve blood pressure control and medication adherence, and the control group receives standard care offered by the treating hospital's outpatient clinics and primary care provided by the participants' general practitioner. Data are collected at four time points: enrolment baseline data collection (T1), immediately following delivery of the intervention (T2), three months post-intervention (T3) and 9-months post-intervention (T4).

### Study aims

The primary aim of the study were to evaluate the effectiveness of a multifactorial intervention in terms of improved systolic blood pressure control in participants with co-existing diabetes and chronic kidney disease. Secondary aims were to determine the effectiveness of the intervention on medication adherence, medication self-efficacy and general wellbeing in consumers with co-existing diabetes and chronic kidney disease. The extent of the relationship between pill counts, self-report of medication adherence and self-efficacy, and other clinical and surrogate biochemical markers of disease control will also be evaluated.

### Study hypotheses

1. Compared to participants receiving standard care, participants with diabetes and chronic kidney disease who receive the intervention will show greater reduction in blood pressure at three months post-intervention (T3).

2. Compared to participants receiving standard care, participants with diabetes and chronic kidney disease who receive the intervention will show a greater adherence to their prescribed medication regimen at three months post-intervention (T3).

3. Improved medication adherence will be associated with increased systolic blood pressure control at three months post-intervention (T3).

### The intervention

Information derived from the literature and other components of the study were used to develop a multifactorial intervention, the *Me*dicine *S*elf-*M*anagement *I*ntervention (MESMI) to improve blood pressure control and medication adherence. The MESMI consisted of self-monitoring of blood pressure, an individualised medication review, a twenty-minute Digital Versatile Disc (DVD), and fortnightly follow-up telephone contact for 12 weeks to support blood pressure control and optimal medication self-management. All components of the intervention were delivered by a renal specialist nurse with doctoral qualifications trained in motivational interviewing using a checklist and standing script for fidelity purposes.

### Self-monitoring of blood pressure

The intervention nurse taught participants in the intervention group to take their own blood pressure using the self blood pressure measurement (SBPM) guidelines of O'Brien et al. [17] and a clinically validated A&D Medical Pty. Ltd. digital blood pressure monitor (Model UA-787, Japan). Participants were asked to take their blood pressure seated every morning on their non-dominant arm at around the same time after waking before they had their morning antihypertensive medications. Participants were informed that their blood pressure may be higher at this time as they had not yet had their antihypertensive medications for the day. Participants were provided with a booklet to record their daily blood pressures for approximately three months.

### Medication review

The individualised medication review involved the intervention nurse drawing up a chart of the participant's prescribed medications as documented by the research assistant at the enrolment visit (T1), which included the generic and brand name of the medication, what the medication was for, the dose and when to take it, and targets for which to aim. Targets included a blood pressure ≤130 mmHg, blood glucose of < 7 mmol/L, Hb A_1C _< 7%, and low density lipoprotein (LDL) cholesterol < 2.5 mmol/L as recommended by Harris [18]. Prescription reconciliation, the process of comparing the participant's reported medication regimen to all of the medications that the participant has been taking, was conducted. Any areas of ambiguity with what participants thought they should be taking compared with what was prescribed according to pill containers and medication lists were sorted prior to obtaining agreement of the prescription. For example, one participant was taking duplicate cholesterol-lowering drugs that were the same but had different brand names. The medication chart was left with the participants as their personal medication record to take to medical consultations if they did not have a current medication list, and to note changes in their prescriptions as they occurred.

### DVD development

The DVD involved an interactive, psychosocial approach to motivating people to take their medications, appealing to knowledge, thoughts and feelings, underpinned by the modified Health Belief Model [15]. By focusing on the attitudes and beliefs of individuals, we believed a person would be more likely to take their medications as prescribed if they understood the inter-relationship of their conditions, in particular hypertension, and if their confidence in independently managing their health was improved.

The content of the DVD was drawn from analysis of medication adherence from the perspectives of consumers with co-existing diabetes and chronic kidney disease and health professionals likely to care for this group [19]. Knowledge of these aspects is likely to facilitate effective communication between consumers and health professionals, and contribute to medication safety and improved wellbeing [20]. The DVD comprised three sections: how blood pressure affects the body; the need, benefit and safety of prescribed medications; and tips to help consumers take their medications as prescribed. Video clips of participants with co-existing diabetes and chronic kidney disease not involved in the RCT who were willing to share their experiences with taking multiple medications were incorporated into each section. These participants talked about how they managed their medication regimens on a daily basis to facilitate medication adherence using a psychosocial approach. As a result it was hypothesized that participants in the trial would control their blood pressure and learn effective medication self-management skills.

The content of the DVD was checked against information in the public arena to ensure a consistent message was being conveyed. We also made sure that the text could easily be read by consumers with visual impairment (≥ 24 Arial font) and understood at a Year 8 level. The DVD was developed under guidance of the project team and a reference group drawn from the key stakeholders of participants and health professionals engaged in the research.

The DVD underwent formative evaluation for internal validity by nine health professionals, one educational expert, and one consumer who provided video footage. Content, readability (flow, clarity, language, layout), and appropriateness of information to meet the learning objectives and desired outcomes were critically evaluated using the Content Validity indices [21], which were respectively 0.98, 0.95 and 0.97. These values exceeded the parameter of 0.83 for item acceptability. Overall, comments were very positive, and included constructive editorial suggestions. Advice was also obtained on screen design features and navigation of the interface, which were incorporated into the final version. The DVD was produced by the university's biomedical multimedia unit incorporating educational design expertise.

### Motivational interviewing telephone calls

Fortnightly telephone calls were planned after the intervention home visit until follow-up at 3-months post-baseline (T2). Each call was conducted using the principles of motivational interviewing by the intervention nurse to promote medication adherence, adapted from the guidelines of Dilorio et al. [22]. The telephone call commenced with an open-ended question inquiring about the participants' wellbeing. An enquiry about their blood pressure followed, whereby participants reported their blood pressure readings for discussion. A general enquiry was then made about their medications, such as whether any changes were made to their medications, if they experienced any side effects or difficulties taking their medications, if they have any concerns with doctors' visits and if they had any questions regarding their health.

The participants' motivation and confidence in taking their medications was then assessed out of a score of 1 to 10, using this information to elicit barriers, concerns and positive self-motivational statements. A summary of these comments was presented back to the participant, inviting any further comments using reflective and normalising statements where appropriate to elicit problem-solving. The participants was then asked to identify three goals they would like to achieve in the next five years to help link their medication adherence to their life. They were also asked to briefly explore connections between their current medication-taking behaviour and their ability to achieve these goals. A summary was presented back to the participants, incorporating suggestions to overcome identified difficulties and affirming desired behaviour. The participants were asked if they had any queries which were addressed, and the call was concluded after thanking the participants for their time and interest. The remaining motivational interviewing calls followed a similar format that incorporated follow-up of suggestions or queries made in previous calls.

### Usual care

Participants randomized to the usual care control group received standard care offered to patients with co-existing diabetes and chronic kidney disease attending the diabetes and nephrology outpatients' clinics at the hospital and primary care offered by their general practitioners. Our definition of standard care is the ongoing medical care provided to a patient with co-existing diabetes and chronic kidney disease attending routine outpatient clinic follow up, which has been reported elsewhere [23]. Data were collected from the usual care group at the same time points as the intervention group.

### Feasibility of study

In the planning stages of the study in 2006, we found that more than 1,400 patients with diabetes at the hospital where recruitment was to take place had urine microalbumin/creatinine ratios ranging from 2 to an extreme of 6020 mg/mmol, with an average microalbumin/creatinine ratio of 97 mg/mmol suitable for our recruitment purposes. Additionally, the renal department at the hospital is one of the largest renal services in Australia treating in excess of 2,300 patients annually with chronic kidney disease. The base rate of systolic blood pressure control > 130 mmHg in the nephrology outpatient clinic population was estimated to be about 50%.

### Setting and recruitment

A research assistant who was a qualified registered nurse recruited from the diabetes and nephrology outpatient clinics of a metropolitan hospital in Melbourne commencing in August 2008. This research assistant conducted all data collection for the study and was a different person to the intervention nurse. There were two diabetes clinics per week for patients requiring ongoing review with approximately 50 patients booked in per clinic. If the patient did not have hypertension, they were immediately deemed ineligible irrespective of the presence of renal disease. There was one renal clinic per week with approximately 50 patients booked in of whom about 15 had either type 1 or type 2 diabetes. Participants were also recruited from a combined diabetes and nephrology clinic (NephDiab clinic) established to incorporate both diabetes and nephrology specialist review within the same appointment where approximately 20 patients were booked in every month. Patients who met the inclusion criteria were approached by the research assistant and asked if they wanted to participate in a study designed to help them understand and manage their complex medical conditions and prescribed medications better.

### Patient eligibility

The inclusion criteria for the MESMI study are listed in Table 1. The primary outcome was systolic blood pressure control, taken by the research assistant who was trained to take blood pressure according to accepted guidelines [17] using an Accosson mercury desk sphygmomanometer and 3M™ Littmann classic 11 S.E. stethoscope. Participants were to avoid smoking, exercise and caffeine for 30 minutes while they were waiting for their clinic consultation and were seated for approximately five minutes prior to having their blood pressure measured.

**Table 1:** Inclusion and exclusion criteria for the MESMI study

Inclusion criteria	
*	Patients of either gender aged ≥ 18 years of age
*	Able to comprehend English (English proficiency test > Not at all)
*	Mentally competent (Abbreviated Mental Test score > 6)
*	Type 1 or Type 2 diabetes recorded in patient's medical record
*	Chronic kidney disease defined by an estimated Modified Diet in
*	Renal Disease glomerular filtration rate (MDRD-eGFR) of ≤ 60 mL/min/1.73 m^2 ^and/or:
*	Diabetic kidney disease defined by Malb/Cr ratios > 2.0 mg/mmol for men, > 3.5 mg/mmol for women or an elevated 24 hour urine albumin of > 30 mg over 24 hours
*	Systolic hypertension ≥130 mmHg for the previous two clinic visits & prescribed antihypertensive medication/s
	

**Exclusion criteria**	

*	Impending dialysis defined by an eGFR less than 15 mL/min/1.73 m^2^
*	Pregnancy
*	A new diagnosis of cancer
*	A mental illness not stabilized with medication

The participant's blood pressure was taken by the research assistant five times at recruitment, commencing with once on either arm in the sitting position. The blood pressure measurement was then repeated twice on the arm with the lower systolic blood pressure at one minute intervals. These two blood pressure readings were then averaged, and the mean measure was used to check the presence of hypertension. Finally, the standing blood pressure was then taken on the same arm within three minutes to detect the presence of orthostatic hypotension, a common side effect of antihypertensive medications in the older population [17].

Consumers who were not mentally competent (assessed by a score of < 6 on the Abbreviated Mental Test) [24], had an unstable mental illness, or did not understand the English language sufficiently were excluded. Proficiency in spoken English was assessed by asking participants from non-English speaking backgrounds how well they spoke English using a four-point scale ranging from very well to not at all [25]. Persons who spoke English not well who had a support person with a good command of the English language were included whereas persons who scored 'not at all' on the Proficiency in Spoken English Test, irrespective of the presence of support persons who spoke English, were excluded from the trial.

### Ethics approval and data and safety monitoring

Prior to commencing the study, ethics approval was sought and granted from the Human Research Ethics Committees of the participating hospital (HREC Project No: 2006.239) and university (Ethics ID: 0713622). The trial was prospectively registered with the Australian and New Zealand Clinical Trials Register (ANZCTR) before recruitment of the first participant (Registration number ACTRN12607000044426). Written informed consent was obtained from participants prior to enrolment. The study was conducted according to the protocol and under ethical guidelines of the National Health and Medical Research Council of Australia [26]. Regular weekly meetings were held between members of the research team to discuss issues relating to the progress of the trial.

### Enrolment and randomization

Participants who expressed an interest in participating and who met the inclusion criteria were contacted by the research assistant by telephone to organize a home visit. At the home visit, formal informed consent was obtained prior to collecting demographic, social and medical data, sitting blood pressure according to accepted guidelines [17] (taken twice on the arm with the lowest reading a minute apart and averaged), body mass index, comorbidity burden, baseline surveys and pill counts (Table 2). The baseline visits lasted approximately 1 1/4 hours each.

**Table 2:** Schedule of data collected from consumers at each data collection point

Measures	T1	T2	T3	T4
Demographics(age, gender, education, usual role, social support)	X			
English proficiency	X			
Abbreviated mental health test	X			
Chronic conditions	X			
Prescribed medications	X			
Body Mass Index	X			X
Blood pressure	X	X	X	X
SF-12v2-Item Health Survey	X	X	X	X
MASES Self-Efficacy Scale	X	X	X	X
Morisky scale	X	X	X	X
Health care utilisation scale	X	X	X	X
Pill count	X	X	X	X
Serum & urine laboratory measures ie eGFR, urine Malb/Cr ratios, creatinine, Hb, HbA_1C_, CaPO4, lipids	X	X	X	X

All participants have data collected at four points in time (Table 2): baseline (T1), 3-month following the baseline data collection and at completion of the intervention (T2), and 6- (T3) and 9-months (T4) post-intervention (12-months post-baseline data collection). Routine clinical laboratory surrogate measures indicative of medication adherence taken as part of the participant's standard treatment such as eGFR, urine microalbumin/creatinine ratios, serum creatinine, Hb, Hb A_1C_, CaP04 and low-density lipids are collected at these four time points from the participant's medical records. Follow-up is projected to continue until July 2010.

### Instruments

The Morisky Medication Adherence Scale [27] was used to measure adherence to all prescribed medications, has four questions with 'yes' and 'no' responses and a Cronbach's α coefficient of 0.62. The Medication Adherence Self-efficacy Scale (MASES) [28] has 26 questions identifying difficult situations affecting medication self-efficacy, with 'not at all sure', 'somewhat sure' and 'very sure' answers, takes about five minutes to complete and has a Cronbach's α coefficient of 0.95. The Short Form (SF)-12, 12 item Health Survey (version 2) [29], was used to measure general wellbeing, has five choices of responses and takes approximately two minutes to complete. Cronbach's α coefficients for the SF-12 closely mirror that of the SF-36 at 0.89 and 0.76. The Health Care Utilization (HCU) [30] Scale has four items of health care, for example, visits to a doctor, where participants have to indicate the number of times they used the service in the past three months, and takes approximately two minutes to complete. The HCU has a test-retest reliability of.0.91 [9].

### Pill counts

Pill counts were conducted at T1, T2, T3 and T4 time points according to the process outlined by Haynes et al. [31]. At enrolment, participants were asked to keep their empty pill containers for the duration of the study. On each data collection home visit, participants were invited to present all their prescribed medications which were manually counted. The prescribed regimen off the container, the dispensing pharmacy, the number of tablets dispensed and the dispensing date for all tablets was recorded. The participants were asked if they kept their medications anywhere else (for example, a duplicate supply), and these were also counted and recorded. The participants were asked if they started taking pills from the containers on hand on the dispensing date, or if not, when they did start taking the medication from the container.

Pill counts were estimated by dividing the number of pills missing from the containers by the number of pills which should be missing if the participant had taken all medications as prescribed [31]. This figure was then multiplied by 100 to obtain a percentage score. For example, Adherence A = (# of pills taken/#of total doses) × 100. When more than 100% was calculated, Adherence B was calculated, which was: Adherence B = 100 - (Adherence A -100): if Adherence A was 120%, Adherence B was 80% [32].

Medication adherence was set at ≥80% as the minimum acceptable level for each prescribed medication, a commonly accepted cut-off point for defining a therapeutic level of medication adherence [33]. Insulin with different devices was hard to quantify when we did not have the resources to buy equipment to weigh the insulin. We decided to use serum blood glucose, HbA_1C _and evidence of hypoglycaemic and hyperglycaemic episodes requiring hospitalization as evidence of medication nonadherence.

### Random assignment and blinding

Following the baseline visit, a statistician off-site conducted the centralized, stratified block randomization of participants according to age, gender and baseline systolic blood pressure to maintain allocation concealment and to prevent selection bias. The identity of all participants who were randomized to receive the intervention was kept in a locked cabinet in the leading researcher's locked office according to university and hospital ethics approval. Data collection conducted by the research assistant was blinded to the intervention group. At the 9-month visit (T4), participants will be asked if they think they received standard care or the intervention or were unsure and why [34]. However, participants in the intervention group could not be blinded as the intervention was delivered face-to-face and it would be expected that this group would accurately guess their group assignment. Participants in either group received no remuneration for participation.

### Power analysis

We based our sample size on Gerin et al.'s [35] medication adherence and blood pressure control (ABC) research, using the standard deviation for systolic blood pressure of 12.1 mmHg cited in the usual care systolic blood pressure group. The sample size determined to allow the detection of a mean (± SD) difference in systolic blood pressure of 3-6 mmHg between the intervention and control groups. The sample size calculation has yielded 51 participants per group with 80% power (a = 0.05 [one-tailed], including 5% attrition, totalling 108 participants in all.

### Participant flow

**Figure 1:**
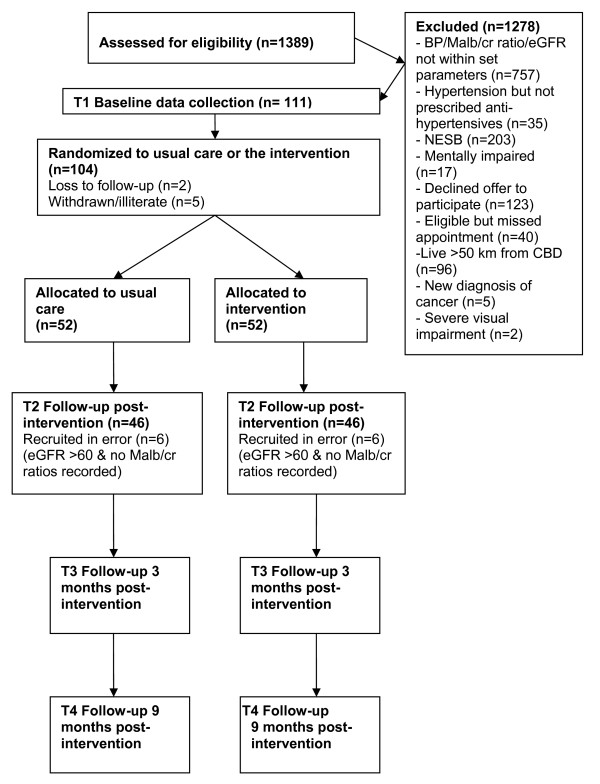
**Trial participant flow**.

Recruitment commenced on the 27th August 2008 and ceased on 17^th ^June 2009. A total of 111 participants were recruited and baseline data taken. The enrolment baseline visits commenced on the 8^th ^September 2008 and ceased on 22^nd ^June 2009. Participant flow is shown in Figure 1.

### Data analysis

The analysis will be performed on an intent-to-treat basis. All data will be entered into an ACCESS database by a second research assistant who is blinded to the intervention group. An experienced biostatistician will check the integrity of the database and conduct statistical analyses. Descriptive statistics will be used to summarise demographic, social and medical data between the intervention and usual care groups and any effect of these variables on primary and secondary outcomes. Baseline characteristics of the intervention and usual care groups will be compared. Blood pressure, medication adherence, medication self-efficacy, and general wellbeing between the usual care and intervention groups will be compared to assess the effects of the intervention between groups and within participants at T2, 3 and 4 using t-tests as appropriate. The various measures of medication adherence will be examined for correlations. Continuous variables that are not normally distributed will be examined using equivalent non-parametric tests. Differences in change in binary categorical variables within each group will be analysed using McNemar's test and the changes between the two groups will be assessed by chi-squared tests for trends. Potential confounders may include comorbidities and number of prescribed medications. Statistical significance will be determined with p values of < 0.05.

## Discussion

This trial involves an evaluation of the effectiveness of a multifactorial intervention developed from analysis of medication adherence from the perspectives of consumers with co-existing diabetes and chronic kidney disease and multidisciplinary health professionals likely to be involved in their management. This approach was chosen to foster effective communication and partnerships with health professionals necessary for improved medication adherence and patient health outcomes. The major strength of this trial is that it is one of the first published studies to tackle the problem of competing comorbidity management where consumers are required to consult with multiple health practitioners and take multiple prescribed medications.

### Recruitment difficulties

The inclusion criteria presented difficulties, particularly in relation to pathology results recorded in the patient's electronic or hard copy of their medical record to determine eligibility. Although patients had diabetes and evidence of chronic kidney disease, patients did not routinely have microalbumin/creatinine ratios or less commonly, 24 hour urine albumins taken. Additionally, some estimated glomerular filtration rates were recorded as '≥ 60' or '< 90' depending on the point-of-care, and if patients did not have a microalbumin/creatinine ratio recorded in their history, they were excluded. Therefore, we have recruited participants with co-existing diabetes (Type 1 or 2) and microalbumin/creatinine ratios > 2.0 mg/mmol for men, > 3.5 mg/mmol for women or co-existing diabetes (Type 1 or 2) and chronic kidney disease (eGFR ≤ 60 mL/min/1.73 m^2^). The development, dissemination and implementation of chronic kidney disease clinical practice guidelines that harmonize with other specialties are needed to improve patient outcomes of chronic kidney disease [36]. We also changed the inclusion criteria to systolic hypertension ≥130 mmHg for the previous clinic visit rather than the previous two clinic visits to improve the rate of recruitment. These deviations and rationales have been documented with the ANZCTR. Our experience has shown that it is essential to pilot recruitment when funding limitations constrain the time period to run a RCT.

Another unforeseen problem affecting recruitment was a change in the organization of the diabetes outpatient clinics. Since commencing the study, patients with diabetes were 'handed over' to their general practitioners to manage in primary care rather than attend routine three monthly hospital outpatient clinic follow-up (these outpatient clinics were reduced to annual review), effectively reducing the pool of patients from which to recruit.

At recruitment, we found a significant number of participants spoke a language other than English, most commonly Greek and Italian, who were excluded from the trial because they did not speak English. The latest Australian census shows that one quarter of Australians are born overseas [37], and consumers from culturally and linguistically diverse backgrounds have an increased risk of medication mismanagement [38]. In addition, 123 patients who met the inclusion criteria refused the opportunity to participate and a further 40 patients who met the inclusion criteria did not turn up for their scheduled appointment. A run-in period would have alerted us to these difficulties and enabled us to introduce strategies to enhance recruitment, such as clinic appointment reminders and reimbursement for participation.

### Assessing medication adherence

Another problem was objective evaluation of medication adherence. The cost of electronic pill monitoring for multiple medications was prohibitive. Difficulties with manual pill counts included the sheer number of medications that the participants had on hand. Very few participants could recall when they started their new prescriptions. Some participants were found to have six boxes of the same medications, all of which had been partially used. In another case, a husband and wife shared their prescribed medications, upsetting the count. Many had different storage areas for medications that were not currently prescribed, in addition to complementary medications. There were discrepancies between what participants said verbally and what was being counted by the research assistant. For example, participants stated that they had not missed any pills but their scripts had ran out, and they were found to have missed at least one or two pills or there were more medications counted that should have been taken. One participant admitted to fiddling with her medications, and took gliclazide 80 mg and gliclazide MR 30 mg on alternate days as she was not sure 'which one was better'. Participants also decanted from one container into another. In one instance, there were 65 pills in a container that should have had 50 pills in it. Our research has emphasized the need for an objective, affordable method to monitor adherence to multiple prescribed medications.

Despite these issues, the management of comorbidities is an increasing component of health care practice, and as such, is in need of interventions that improve patient outcomes. This research has highlighted the need for quality medical record documentation and a systematic, evidence-based approach to patient care that overrides the needs of specialties in the patient with competing diagnoses.

## Competing interests

The authors declare that they have no competing interests in the conduct of this research. The funding source had no involvement in the study design and methods.

## Authors' contributions

AW, EM & RW: (a) conception and design, or acquisition of data, or analysis and interpretation of data; and (b) the drafting of the article or critical revision for important intellectual content and c) final approval for the version to be published.

## Pre-publication history

The pre-publication history for this paper can be accessed here:

http://www.biomedcentral.com/1471-2296/11/3/prepub
